# Achieving High Strength–Ductility Synergy in Low-Alloyed Mg–Li–Er Extrusion Alloys via Tailoring Bimodal-Grained Structure

**DOI:** 10.3390/ma17143506

**Published:** 2024-07-15

**Authors:** Ruyue Tang, Jing Zhang, Zhenwei Gong, Bingcheng Li, Quan Dong

**Affiliations:** 1 College of Materials Science and Engineering, Chongqing University, Chongqing 400044, China; 2 National Engineering Research Center for Magnesium Alloys, Chongqing University, Chongqing 400044, China

**Keywords:** Mg alloys, extrusion, bimodal grain structure, tensile property, strengthening mechanism

## Abstract

Low-alloyed Mg–Li–Er alloys were developed in this study and a bimodal-grained structure was obtained by varying the trace Er content and extrusion temperature. The alloys displayed a good strength–ductility synergy, i.e., a tensile yield strength (TYS) of 270 MPa and an elongation (EL) of 19.1%. Microstructural characterization revealed that the formation of numerous submicron Mg_24_Er_5_ particles favored a high density of low-angle grain boundaries (LAGBs) inside the deformed grains and inhibited dynamic recrystallization (DRX). The resultant coarse unDRXed grains with a strong basal texture and considerable LAGBs, together with the fine DRXed grains, contributed to the high strength–ductility synergy.

## 1. Introduction

Mg alloys have attracted extensive attention in the aerospace and automobile fields due to their low density, high specific modulus, and ease of recycling. However, unlike other structural materials’ mechanical properties, Mg alloys have a poor ductility and inadequate strength [1,2]. Numerous theoretical studies [3,4,5] have indicated that Li alloying effectively improves this ductility by activating non-basal slip by decreasing the c/a ratio or critical resolved shear stress (CRSS) ratio between the non-basal slips and basal slip. Generally, Mg-Li alloys have a good ductility, as well as lightweight advantages [6]. However, their inadequate strength greatly limits the widespread application of Mg–Li alloys. Therefore, developing Mg–Li alloys with a high strength–ductility synergy will be an effective measure to increase the industrial use of Mg alloys.

Alloying is an important strategy to improve the mechanical properties of Mg alloys, especially the alloying of rare earth (RE) elements [7,8]. Among RE elements, Erbium (Er) has received much attention due to its significant property improvement effect. On the one hand, Er can refine grains and improve the strength of alloys, and it is noteworthy that Er has an obvious grain-refining effect in Mg–Li alloys [9]. Moreover, due to its poor atomic diffusion ability, Er delays the recrystallization process and improves the high-temperature strength and creep resistance of Mg alloys [10,11]. On the other hand, adding Er is favorable for improving the alloy’s ductility. For instance, Zhang et al. [12] fabricated a diluted Mg–0.3 at% Er alloy with a surprisingly ultrahigh ductility (nearly 50%) at room temperature (RT). They reported that trace Er elements can activate a large number of non-basal slips. This point was also confirmed by our previous work [13]. In addition, we found that Er microalloying can enhance intergranular coordination.

Recently, many studies have reported that forming bimodal-grained structures could favor a desirable strength–ductility synergy. For example, Mg alloys with bimodal-grained structures, such as Mg–Zn alloys [14,15] and Mg–Gd alloys [16,17], exhibit a high yield strength and maintain a relatively high ductility. It is widely known that the formation of bimodal-grained structures is strongly associated with the DRX process [18,19]. Generally, the extrusion process and the alloying of elements’ content conditions are two key factors affecting DRX behavior [20]. Sun et al. [21] studied Mg–3.0Zn–0.2Ca–0.5Y alloys with different extrusion temperatures, and the experimental results showed that multimodal-grained alloys (sample extruded at 250 °C) had a high strength and ductility compared to their fine-grained counterparts (sample extruded at 300 °C). In addition, Li et al. [22] compared the microstructure and mechanical properties of AZ91 and AZ91–1Y alloys. They reported that coarse Al_2_Y particles lead to inhomogeneous recrystallization, while dispersed submicron Mg_17_Al_12_ precipitates inhibited the growth of recrystallized grains. Therefore, it is considered that bimodal-grained structures can be tailored by utilizing proper extrusion process parameters and alloying element contents.

Based on the design strategy above, low-alloyed Mg–3Li–*x*Er (*x* = 0.2, 0.8 wt%) alloys were developed in this study. Different microstructural characteristics were tailored by changing the trace Er content and extrusion temperature. In addition, the underlying strengthening mechanisms regarding bimodal-grained structures were discussed in detail. The results provide essential knowledge on the effects of alloying elements and the deformation temperature on the bimodal-grained structure, and offer an efficient method for developing low-alloyed Mg alloys with a high strength–ductility synergy.

## 2. Experimental Procedure

Pure Mg (99.97 wt.%), Li (99.90 wt.%), and Mg-25 wt% Er master alloys were utilized to prepare Mg–3 Li–*x* Er (*x* = 0.2, 0.8 wt%) alloys, and the raw materials were supplied by Chongqing Yuhua New Material Technology Co., Ltd. (Chongqing, China). The practical compositions of the alloys measured by inductively coupled plasma optical emission spectroscopy (ICP-OES, Optima 8000, PerkinElmer, Waltham, MA, USA) were Mg–3.01, Li–0.22 Er, and Mg–3.02 Li–0.78 Er (wt%), respectively. The as-cast alloys were homogenized at 300 °C for 12 h and then quenched in water. After preheating at 300 °C for 30 min, the billets were extruded by the XJ-500 horizontal extruder (Wuxi Yuanchang Machine Manufacture Co., Wuxi, China) at 260 °C and 300 °C with an extrusion speed of 2 mm/s and an extrusion ratio of 25:1. The specimens were named for ease of description, e.g., the Mg–3Li–0.2Er alloy extruded at 260 °C is called LE302–260 hereafter. The dog-bone-shaped tensile specimens were sampled longitudinally with 16 mm × 4 mm × 2 mm dimensions. The specimens were tensile loaded to failure at room temperature by the CMT5105 universal test machine (MTS Systems Co., Ltd., Shanghai, China) at a rate of 1 mm/min, and three parallel samples were tested to ensure accuracy.

The microstructures were observed by an optical microscope (OM, ZEISS Axiovert, Jena, Germany) and scanning electron microscopy (SEM, JSM-7800F, JEOL Ltd., Tokyo, Japan) equipped with energy dispersive spectroscopy (EDS, 80 mm^2^ X-Max^N^ Silicon Drift Detector, Oxford Instrument, Oxford, UK) after etching with ethylene glycol acetate solution (1% nitric acid, 20% acetic acid, 60% ethylene glycol, and 19 vol.% deionized water). The volume fraction and diameters of the second phases and the recrystallization fraction were counted using Image-Pro Plus 6.0 software, and the average grain sizes were measured using the linear intercept method. The as-extruded specimens were examined by electron backscatter diffraction (EBSD, NordlysMax^2^ detector, Oxford instrument, UK) equipped with a HKL EBSD system at 20 kV with a step size of 0.5 μm, after grinding and electro-polishing in ethanol −5% perchloric acid electrolyte for 240 s with a direct voltage of 20 V and temperature of −30 °C. The corresponding EBSD data were processed by Channel 5.0 software to obtain pole figures, low-angle grain boundaries (LAGBs), grain sizes, etc. The ranges of the angular differences between the grain boundary and sub-grain boundary orientations were chosen to be 15°~90° and 2°~15°.

## 3. Results and Discussion

### 3.1. Microstructure Characterization

**Figure 1:**
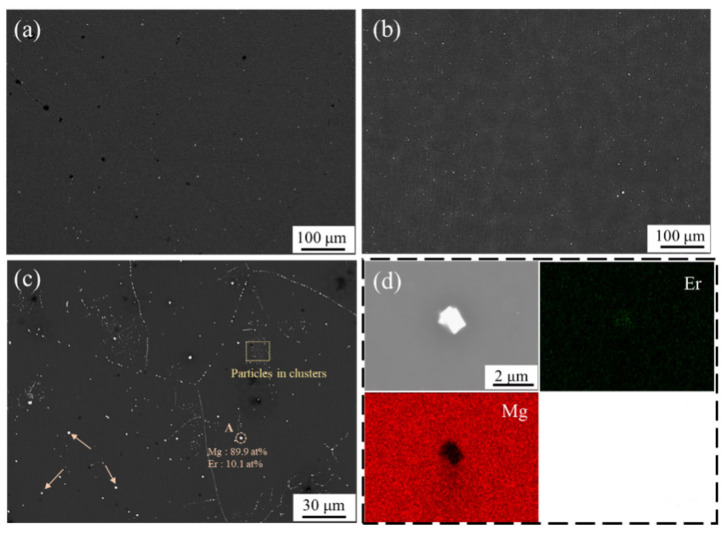
SEM images of the as-homogenized Mg–3Li–0.2Er alloy (**a**) and Mg–3Li–0.8Er alloy (**b**) at low magnification. Higher-magnification SEM image and particle EDS analysis result (**c**) and EDS mappings (**d**) of Mg–3Li–0.8Er alloy.

Figure 1a–d show the SEM images and EDS results of the as-homogenized Mg–3Li–0.2Er alloy and Mg–3Li–0.8Er alloy. Figure 1a,b display the volume fraction of the particles in the two alloys at a low magnification, with very few bright white particles in the as-homogenized Mg–3Li−0.2Er alloy. Comparatively, the amount of particles in the α-Mg matrix of the Mg–3Li–0.8Er alloy (Figure 1b) was slightly higher. Two size scales of particles can be observed in the higher-magnification SEM image (Figure 1c), with the coarse phase uniformly distributed in the α-Mg matrix and submicron particles clustered together, labeled with arrows and boxes, respectively. The compositions of particle A and the corresponding EDS mapping analysis are shown in Figure 1c,d, respectively, indicating that these particles can be clearly identified to be Mg_24_Er_5_ phase enriched with 89.9 at.% Mg and 10.1 at.% Er elements. In addition, according to the EDS mapping, it can be seen that most Er was dissolved into the Mg matrix, and a few Er elements existed in the form of compounds.

**Figure 2:**
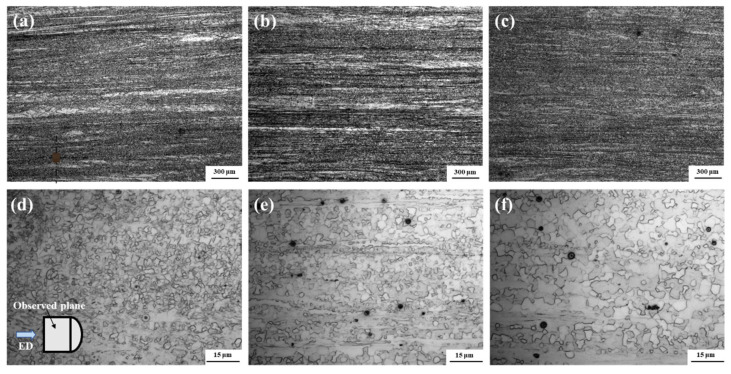
OM images of the as-extruded Mg–3Li–*x*Er (*x* = 0.2, 0.8) alloys at different magnifications ((**a**–**c**) low and (**d**–**f**) high magnifications): (**a**,**d**) LE302−260; (**b**,**e**) LE308–260; and (**c**,**f**) LE308–300.

Figure 2a–f show the different magnification OM images of the as-extruded Mg–3Li–*x*Er (*x* = 0.2, 0.8) alloys at different extrusion temperatures. The microstructure of the alloys can be observed alone on the ED-ND plane, as shown schematically in Figure 2d. The LE302–260 and LE308–260 alloys exhibited a bimodal-grained structure distribution consisting of fine DRXed grains and coarse deformed grains, which were in the range of 20–70 μm, indicating that incomplete recrystallization occurred in the Mg–3Li–*x*Er (*x* = 0.2, 0.8) alloys extruded at 260 °C. Compared to the Mg–3Li–0.2Er alloy (Figure 2a), the number of coarse deformed grains in the Mg–3Li–0.8Er alloy (Figure 2b) was greater, the size was larger, and the DRX fractions of the two groups were about 87% and 84%, respectively, implying that the minor Er content suppressed the dynamic recrystallization of the Mg–3Li alloy to a certain extent. As the extrusion temperature rose to 300 °C, the Mg–3Li–0.8Er alloy (Figure 2c) was nearly fully recrystallized with the DRX fraction up to 95%. Moreover, the average grain sizes in the DRXed regions of the LE302–260, LE308–260, and LE308–300 alloys were estimated as 3.7 ± 0.44 μm, 3.1 ± 0.38 μm, and 5.5 ± 0.56 μm, respectively.

**Figure 3:**
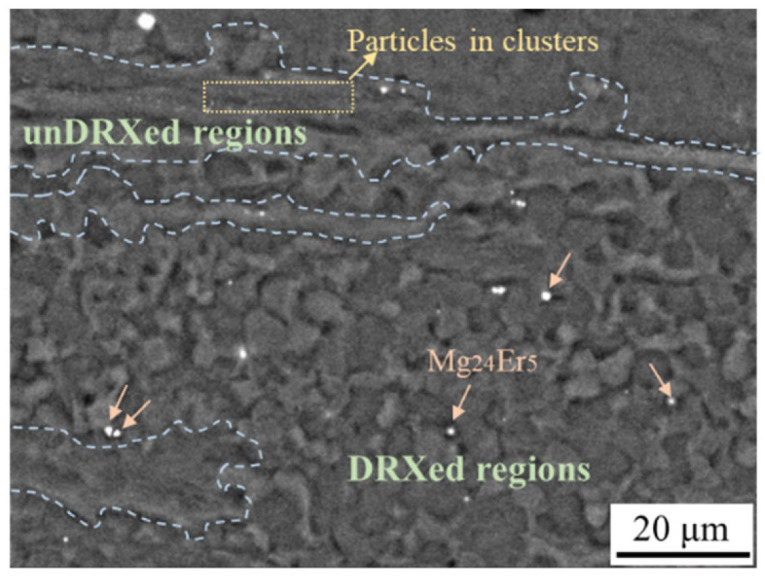
SEM image showing the distribution of the different size scales of particles for LE308–260.

In order to investigate the effect of the different size scales of particles on the bimodal-grained structure, the microstructure of the as-extruded Mg–3Li–0.8Er alloy (LE308–260) was further characterized using SEM, and the distribution of particles is shown in Figure 3. The image clearly shows that the coarse Mg_24_Er_5_ phase was distributed in the equiaxed DRXed region, while the submicron particles aggregated in clusters were distributed in a scattered manner in the coarse unDRXed grains (The dotted lines areas in Figure 3), labeled with arrows and boxes in Figure 3, respectively. It is well known that dynamic recrystallization behavior is affected by the size and distribution of the second phase, which, in turn, determines the final microstructure of the alloys. Humphreys et al. [23] proposed that particles with a diameter larger than 1 μm can act as nucleation sites, which promotes recrystallization because of the higher dislocation density and orientation gradient in its vicinity. On the other hand, submicron particles exert a strong pinning effect on dislocations, delaying the migration of grain boundaries and, thus, inhibiting the recrystallization process [24]. For Mg–3Li–*x*Er (*x* = 0.2, 0.8) alloys, the coarse phase promoted dynamic recrystallization nucleation and facilitated grain refinement, whereas the submicron particles, which were aggregated in the form of clusters, hindered dislocation motion, promoted substructure formation, and slowed recrystallization. According to the SEM images (Figure 1c), the volume fraction of the coarse phase in the Mg–3Li–0.8Er alloy was about 0.32% vol.%, with an average size of 1.63 μm, while the submicron particles with an average size of 420 nm accounted for 2.4% vol.%, which is a large proportion. Based on the volume fractions of the second phase of two size scales, it can be inferred that the minor Er suppressed the dynamic recrystallization, which is consistent with the results obtained from the OM observation.

**Figure 4:**
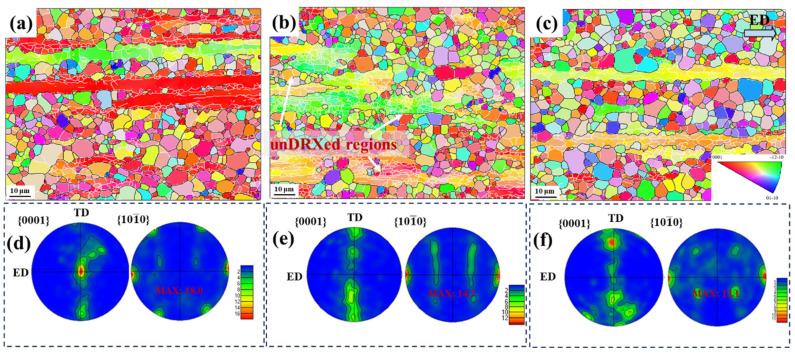
IPF images and PFs of the as-extruded Mg–3Li–*x*Er (*x* = 0.2, 0.8) alloys: (**a**,**d**) LE302–260; (**b**,**e**) LE308–260; and (**c**,**f**) LE308–300.

Figure 4 shows the inverse pole figure (IPF) map and pole figure (PF) of the as-extruded Mg–3Li–*x*Er (*x* = 0.2, 0.8) alloys. Clearly, both alloys consisted of fine recrystallized grains and coarse deformed grains, where the deformed grains were subdivided into sub-grain lamellae by LAGBs (indicated by thin white lines in Figure 4). It can be observed that a small number of LAGBs were inside the deformed grains of the Mg–3Li–0.2Er alloy. Conversely, the amount of LAGBs was significantly higher in the extruded Mg−3Li−0.8Er alloy. In addition, the density of LAGBs decreased significantly as the width of sub-grain boundaries increased with an increase in the extrusion temperature, as shown in Figure 4c. The pole figures (Figure 4d–f) show that the extruded Mg–3Li–*x*Er (*x* = 0.2, 0.8) alloys exhibited a typical (0001)//ED fiber texture, with this extruded fiber texture predominantly derived from deformed grain regions. The maximum texture intensity of the LE302–260, LE308–260, and LE308–300 alloys were 18.0 (mud), 14.7 (mud), and 12.1 (mud), respectively.

### 3.2. Tensile Mechanical Properties

**Figure 5:**
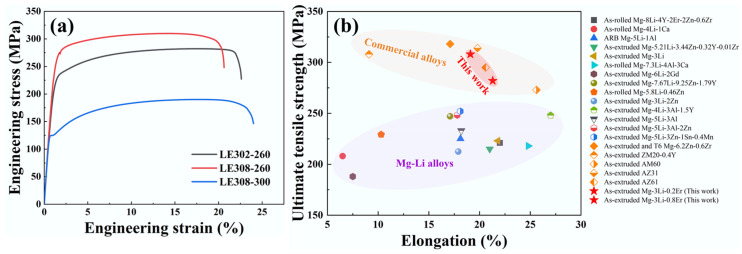
(**a**) The engineering stress–strain curves of Mg–3Li–*x*Er (*x* = 0.2, 0.8 wt%) samples extruded at 260 °C and 300 °C and (**b**) distribution map of UTS and elongation values of previously reported Mg–Li alloys and commercial alloys [25,26,27,28,29,30,31,32,33,34,35,36,37,38,39,40,41,42,43].

Figure 5a shows the tensile engineering stress–strain curves of the Mg–3Li–*x*Er (*x* = 0.2, 0.8 wt%) samples extruded at 260 °C and 300 °C, with the corresponding tensile yield stress (TYS), ultimate tensile stress (UTS), and elongation to failure (EL) summarized in Table 1. It can be seen that the studied alloys extruded at 260 °C displayed a good strength–ductility synergy. The TYS, UTS, and EL of LE302–260 were 225 MPa, 282 MPa, and 21.3%, respectively. Compared to the Mg–3Li–0.2Er alloy, the Mg–3Li–0.8Er alloy exhibited a higher strength (20%) and slightly decreased EL, with the TYS and EL of LE308–260 being 270 MPa and 19.1%, respectively. As the extrusion temperature rose to 300 °C, the ductility of the alloy improved slightly, but the strength decreased dramatically, especially the TYS, by up to 50%. In addition, an apparent yielding phenomenon can be observed in the tensile stress–strain curves of the Mg–3Li–0.8Er alloy and is more pronounced in the LE308−300 specimen. The appearance of the yielding point may be related to the interaction between solute Er atoms and dislocations.

Figure 5b displays the UTS and elongation values of the reported Mg–Li alloys and commercial Mg alloys for comparison. Table 2 shows the state and mechanical properties of the compared alloys in more detail. We can see that the studied alloy system had an excellent strength–ductility synergy, which is significantly better than the mechanical properties of most Mg–Li alloy systems. In addition, the studied alloys are also comparable to some existing commercial Mg alloys, such as AM60 [25], AZ61 [26], and Mg–6.2Zn–0.6Zr [27] alloys. The presented comparative results (Figure 5b) indicate that the Mg–3Li–*x*Er (*x* = 0.2, 0.8 wt%) alloys have the potential to be commercially viable with significant weight reduction advantages.

### 3.3. Strengthening Mechanisms

In this work, the low-alloyed Mg–3Li–*x*Er (*x* = 0.2, 0.8) alloys extruded at 260 °C showed good comprehensive mechanical properties. The microstructure observation showed that the extruded LE302–260 and LE308–260 alloys had a bimodal-grained structure, which is typically characterized by randomly oriented DRXed regions with fine grains, as well as strongly basal-oriented Un-DRXed regions with high-density LAGBs. Thus, based on the rules for mixtures, the average strength of the extruded Mg–3Li–*x*Er (*x* = 0.2, 0.8) alloys can be quantitatively predicted by Equation (1):

*σ*_ys_ = *σ*_1_∙V_1_ + *σ*_2_∙V_2_ (1)

where *σ*_ys_ represents the yield strength of these studied alloys and *σ*_1_ (V_1_) and *σ*_2_ (V_2_) represent the strength and volume fractions of the DRXed and Un-DRXed regions, respectively. 

Hansen pointed out [44] that grain boundaries and dislocations in metallic materials are the main contributors to strain hardening. It is also crucial to express the strength increment induced by the precipitates. In the DRXed regions, the residual dislocation density within the DRXed grains was extremely low (<10^12^ m^−2^) due to the large amount of strain storage energy consumed by the dynamic recrystallization. In the Un-DRXed regions, there were a large number of low-angle grain boundaries and geometrically necessary dislocations (GNDs). Therefore, the yield strength (σ_ys_) in the extruded alloys can be expressed as Equation (2):

*σ*_ys_ = *σ*_GB_∙V_1_ + (*σ*_LAGBs_ + *σ*_dislo_) V_2_ + *σ*_Orowan_ (2)

where *σ*_GB_, *σ*_LAGBs_, and *σ*_dislo_ represent the strength contributions due to GB, LAGBs, and residual dislocations, respectively, and *σ*_Orowan_ represents the Orowan strengthening by precipitates.

**Figure 6:**
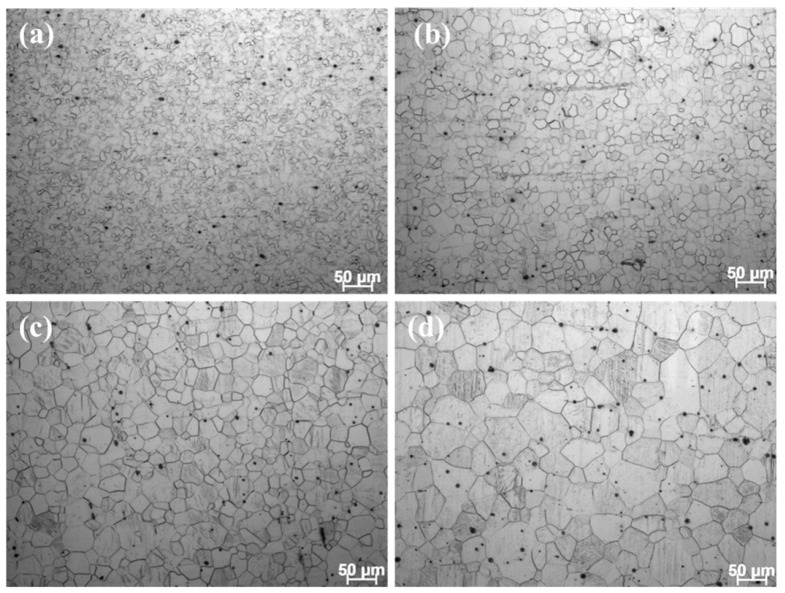
OM images of the as-extruded Mg–3Li–0.2Er alloy after different annealing treatments: (**a**) 350 °C × 1 h; (**b**) 380 °C × 1 h; (**c**) 400 °C × 1 h; and (**d**) 400 °C × 3 h.

**Figure 7:**
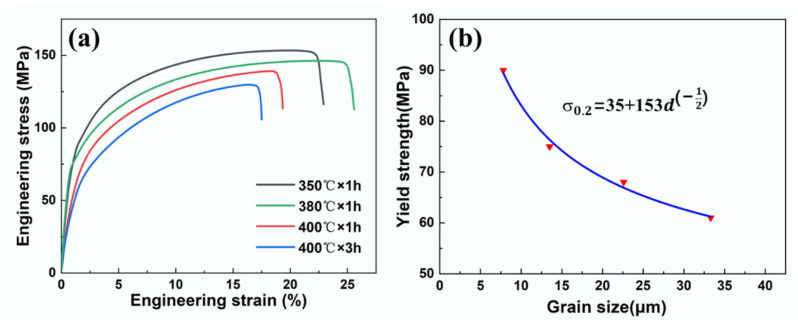
(**a**) The engineering stress-strain curves of as-annealed Mg–3Li–0.2Er alloy and (**b**) Hall–Petch relations of Mg–3Li–0.2Er alloy.

Firstly, the grain boundary strengthening (*σ*_GB_) can be calculated according to the Hall–Petch relation (Equation (3)):

(3) $$\sigma_{GB}=\sigma_{0}+k_{y}d^{-\frac{1}{2}}$$

where *σ*_0_ and *K_y_* are the lattice friction and Hall–Petch coefficient, respectively. It is well known that *σ*_0_ and *K_y_* are intrinsic properties of materials, and they can be significantly affected by the grain size and texture of Mg alloys. To obtain accurate parameters of the Mg–3Li–*x*Er alloys, the LE302–260 specimens were annealed under different treatments to develop fully recrystallized microstructures with four different grain sizes and further tensile testing. Figure 6a–d and Figure 7a show the OM images and engineering stress–strain curves of the as-extruded Mg–3Li–0.2Er alloy after different annealing treatments, respectively. The average grain sizes of the specimens with annealing treatments of 350 °C × 1 h, 380 °C × 1 h, 400 °C × 1 h, and 400 °C × 3 h were determined by the linear intercept method to be 7.8 ± 0.4 μm, 13.5 ± 1.2 μm, 22.6 ± 1.5 μm, and 33.3 ± 2.6 μm, respectively. The yield strengths of these four specimens were 90 MPa, 75 MPa, 68 MPa, and 61MPa, respectively. The *σ*_0_ and *K_y_* were finally determined to be 35 MPa and 153 MPa∙MPa∙μm^−1/2^, respectively, as shown in Figure 7b. For the LE308–300 specimen with a high degree of recrystallization, most of the grains showed a random orientation. Thus, the grain size (5.5 μm) was directly substituted into the Hall–Petch relationship, and the grain boundary strengthening value was calculated to be 100 MPa. 

However, for the LE302–260 and LE308–260 alloys with a strong basal texture, the effect of the texture on the parameters must be considered. In AZ31 alloys with a strong basal texture, Yuan et al. [45,46] found that *σ*_0_ and *K_y_* were 208 MPa and 90 MPa∙μm^−1/2^, respectively, when the grain size was small (<2 μm), while when the grain size was large (>2 μm), *σ*_0_ and *K_y_* were 124 MPa and 205 MPa∙MPa∙μm^−1/2^, respectively. In this work, the texture of the extruded Mg–3Li–*x*Er (*x* = 0.2, 0.8) alloy can be considered as similar to that in Yuan’s work. Therefore, the grain-boundary-strengthening effect can be estimated by neglecting the difference in solute atoms between AZ31 and the studied alloys. For the LE302–260 and LE308–260 alloys, the strength contributions due to grain boundaries were calculated as 230 MPa and 240 MPa, respectively.

In addition, the strengthening of the LAGBs can also be obtained using the Hall–Petch relation, where the average grain size is the thickness of the sub-grain boundaries. The average grain size correction is about ~1.4 times, considering the elongated grain morphology of the sub-grain lamellae [47]. As seen from the IPF (Figure 4), the density of LAGBs within the LE302–260 and LE308–300 specimens was extremely low. Therefore, the strengthening contribution of the LAGBs to the LE302–260 and LE308–300 alloys can be approximately neglected. Conversely, the value of strengthening due to LAGBs in the LE308–260 specimens was calculated to be 262 MPa.

**Figure 8:**
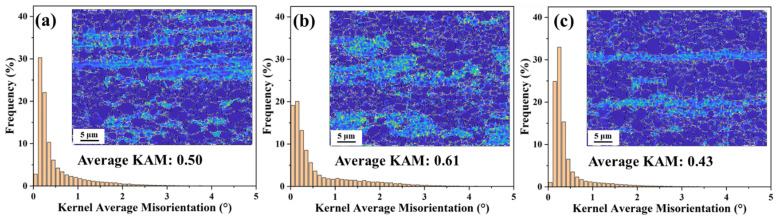
KAM maps and corresponding GNDs density distributions of the as-extruded Mg–3Li–*x*Er (*x* = 0.2, 0.8) alloys: (**a**) LE302–260; (**b**) LE308–260; and (**c**) LE308–300.

Secondly, intense plastic deformation leads to local lattice distortions within deformed grains, which usually generate a high density of residual dislocations to accommodate the local strain, and these dislocations are called “geometrically necessary dislocations” (GNDs). The density of geometrically necessary dislocations (*ρ*_GNDs_) is directly related to the Kernel average misorientation (KAM), which can be obtained using Equation (4) [48]:

(4) $$\rho_{GNDs}=\frac{2\Theta }{ub}$$

where *u* is the unit length, equal to the EBSD step size (500 nm), and *b* is the magnitude of the Parker vector (*b* = 0.32 nm). *Θ* can be determined from the localized orientation distribution in the KAM. Figure 8 shows the KAM maps and the corresponding density distributions of GNDs for the extruded Mg–3Li–*x*Er (*x* = 0.2, 0.8) alloys. The *ρ*_GNDs_ of the LE302–260, LE308–260, and LE308–300 alloys was calculated to be 6.25 × 10^15^ m^−2^, 7.63 × 10^15^ m^−2^, and 5.38 × 10^15^ m^−2^, respectively. The strength contribution from residual dislocations can be determined by Equation (5) [49]:

(5) $$\sigma_{dislo}=M\alpha Gb\sqrt{\rho_{GNDs}}\;$$

where *M* is the Taylor factor (*M* taken as 2.5 for simplicity), α is the constant 0.2, and *G* is the elastic modulus (*G* = 16.6 GPa). Thus, the calculated dislocation strengthening values for the three specimens were 210 MPa, 232 MPa, and 195 MPa, respectively.

As for *σ*_Orowan_ strengthening, since the contents of uniformly distributed micron phases in the Mg–3Li–*x*Er (*x* = 0.2, 0.8 wt%) alloys were very small, only 0.13% and 0.32%, respectively, with an average size of 1.63 μm, the reinforcement brought about by the coarse phase is negligible. The strengthening effect brought about by the submicron spherical particles in the Mg–3Li–0.8Er alloy can be calculated by Equation (6) [50]:

(6) $${\Delta \tau }_{p}=\frac{Gb}{2\pi \sqrt{1-\nu }(\frac{0.779}{\sqrt{f}}-0.785)d_{p}}ln\frac{0.785d_{p}}{b}$$

where Δτ*_p_* is the shear stress, ν is the Poisson’s ratio of Mg alloys (0.35), *f* is the volume fraction of the nanoparticles, and *d_p_* is the average size of the nanoparticles. Thus, the strength contribution from Orowan is estimated to be 16 MPa.

**Figure 9:**
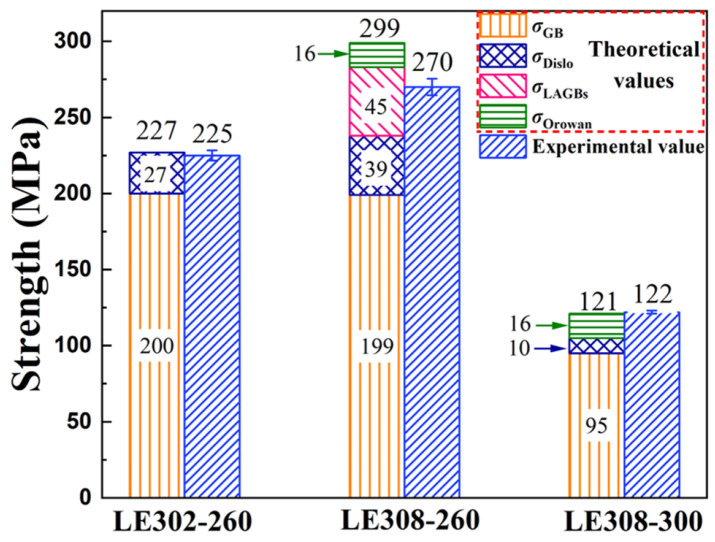
The strengthening mechanisms of Mg–3Li–*x*Er (*x* = 0.2, 0.8) alloys extruded at 260 °C and 300 °C.

Figure 9 shows the strengthening mechanism of the Mg–3Li–*x*Er (*x* = 0.2, 0.8) alloys extruded at 260 °C and 300 °C. Table 3 summarizes the relevant parameters for the strength calculations of the three specimens. The above theoretical calculations give the predicted values of *σ*_ys_ for the LE302–260, LE308–260, and LE308–300 alloys as 227 MPa, 299 MPa, and 121 MPa, respectively, where the predicted values of LE302–260 and LE308–300 specimens are in good agreement with the experimental value. However, the predicted value of LE308–260 was 29 MPa higher than the actual value, largely because of the overestimation of the strengthening effect brought about by the sub-granular boundaries. First, compared with HAGBs, LAGBs with lattice orientation angle differences of less than 15° have a smaller hindering effect on dislocation slip. Moreover, LAGBs consist of dislocation arrays, which will act as a source of dislocation, emitting new moving dislocations [51], so LAGBs do not provide as much reinforcement as HAGBs. In this work, using the Hall–Petch relationship calculation will somewhat overestimate the enhancement effect brought about by LAGBs.

As shown in Figure 9, on the one hand, it is clear that the key to the difference in the YS between the LE302–260 and LE308–260 specimens was the grain-boundary-strengthening effect brought about by the LAGBs. For Mg–3Li–*x*Er alloys, the strengthening effect produced by LAGBs played a vital role, implying that the presence of a submicron second phase is an effective measure to improve the yield strength of alloys. On the other hand, it is worth noting that the comparative results (Figure 5a and Table 1) of the LE308–260 and LE308–300 specimens indicated that the significantly better strength of the bimodal-grained alloys (LE308–260) compared to that of the fine-grained alloys (LE308–300) can be attributed to the superior grain boundary strengthening of the strongly basal-textured alloys.

## 4. Conclusions

In this work, lightweight Mg–3Li–*x*Er (*x* = 0.2, 0.8 wt%) alloys with a high strength–ductility synergy were prepared by simple low-temperature extrusion. The microstructures of the studied alloys were systematically characterized, and the strengthening mechanisms were deeply explored. The main conclusions are as follows:

1. Er existed in the α-Mg solid solution and in the form of a second phase. The second phase had two size scales, with the submicron particles forming clusters inside the grain and the coarse phase evenly distributed in the matrix. The Mg–3Li–0.2Er alloy was almost a single-phase α-Mg solid solution, while there were numerous submicron Mg_24_Er_5_ particles in the Mg–3Li–0.8Er alloy.
2. Minor Er could significantly increase the density of LAGBs during the hot extrusion process and inhibit dynamic recrystallization via a pinning mechanism, which favors developing a bimodal-grained structure.
3. The as-extruded Mg–3Li–*x*Er (*x* = 0.2, 0.8 wt%) alloys with a bimodal-grained structure displayed a good strength–ductility synergy, i.e., a tensile yield strength (TYS) of 270 MPa and an elongation (EL) of 19.1%, which is anticipated to make them suitable for making products for the automobile and electronic industries. Their underlying strength mechanism indicates that their high strength is attributed to the combined effect of the coarse unDRXed grains (CGs) with a strong basal texture and considerable LAGBs, as well as the fine DRXed grains (FGs) with excellent grain boundary strengthening.

## Figures and Tables

**Table 1:** The TYS, UTS, and EL of the Mg–3Li–*x*Er (*x* = 0.2, 0.8 wt%) alloys extruded at 260 °C and 300 °C.

Alloys	Extrusion Temperature (°C)	TYS (MPa)	UTS (MPa)	EL (%)
Mg–3Li–0.2Er	260	225 ± 3	282 ± 2	21.3 ± 0.5
Mg–3Li–0.8Er	260	270 ± 2	308 ± 3	19.1 ± 0.3
Mg–3Li–0.8Er	300	122 ± 1	185 ± 3	23.1 ± 0.4

**Table 2:** The state, UYS, and EL of the previously reported Mg−Li alloys and commercial alloys.

Alloy Systems	Alloys	Stats	UTS (MPa)	El (%)	Refs.
Mg−Li alloys	Mg−−3Li	As-extruded at 300 °C	223	21.8	[28]
	Mg–3Li–2Zn	As-extruded at 300 °C	212	17.9	[28]
	Mg–4Li–1Ca	As-rolled at 300 °C	208	6.5	[29]
	Mg–4Li–3Al–1.5Y	As-extruded at 300 °C	248	27.1	[30]
	Mg–5Li	As-extruded at 300 °C	159	17.0	[31]
	Mg–5Li–1Al	ARB at 350 °C	225	18.1	[32]
	Mg–5Li–3Al	As-extruded at 200 °C	233	18.2	[33]
	Mg–5Li–3Al–2Zn	As-extruded at 300 °C	248	17.8	[34]
	Mg–5Li–3Zn–1Sn–0.4Mn	As-extruded at 200 °C	252	18.1	[35]
	Mg–5.21Li–3.44Zn–0.32Y–0.01Zr	As-extruded at 350 °C	215	21.0	[36]
	Mg–5.8Li–0.46Zn	As-extruded at 200 °C	229	10.3	[37]
	Mg–6Li–2Gd	As-extruded at 300 °C	188	7.5	[38]
	Mg–7.3Li–4Al–3Ca	As-rolled at 350 °C	218	24.8	[39]
	Mg–7.67Li–9.25Zn–1.79Y	As-extruded	247	17.1	[40]
	Mg–8Li–4Y–2Er–2Zn–0.6Zr	As-cold rolled	221	22.0	[41]
	Mg–3Li–0.2Er	As-extruded at 250 °C	282	21.3	This work
	Mg–3Li–0.8Er	As-extruded at 250 °C	308	19.1	This work
Commercial Mg alloys	Mg–6.2Zn–0.6Zr	As-extruded and T6 aging at 160 °C	318	17.1	[27]
	ZM20–0.4Y	As-extruded at 400 °C+T6 aging at 175 °C	308	9.1	[42]
	AM60	As-rolled at 500 °C	273	25.6	[25]
	AZ31	As-extruded at 300 °C	313	19.8	[43]
	AZ61	As-extruded at 400 °C	295	20.6	[26]
	AZ91	As-extruded at 300 °C	350	14.5	[26]

**Table 3:** Relevant parameters for Mg–3Li–0.2Er and Mg–3Li–0.8Er alloys include average grain size (*D*) and the density of geometrically necessary dislocations (*ρ*_GNDs_), as well as the strengthening value from GB, dislocations, and precipitations.

Samples	Regions	GBs Strength		Dislocations Strength		*σ*_orowan_ Strength	Predicted Strength (MPa)
		*D* (μm)	*σ*_GB_ (MPa)	*ρ*_GND_ (10^15^ m^−2^)	*σ*_dislo_ (MPa)	*σ*_orowan_ (MPa)	
LE302–260	Un-DRXed (13%)	-	-	~6.25	~210	-	~227
	DRXed (87%)	~3.7	~230	-	-	-	
LE308–260	Un-DRXed (17%)	~2.2	~262	~7.63	~232	~16	~299
	DRXed (83%)	~3.1	~240	-	-	-	
LE308–300	Un-DRXed (5%)	-	-	~5.38	~195	~16	~121
	DRXed (95%)	~5.5	~100	-	-	-	

## Data Availability

Data will be made available on request.
